# Functional Topography and Development of Inhibitory Reticulothalamic Barreloid Projections

**DOI:** 10.3389/fnana.2018.00087

**Published:** 2018-10-31

**Authors:** Kazuo Imaizumi, Yuchio Yanagawa, Guoping Feng, Charles C. Lee

**Affiliations:** ^1^Department of Comparative Biomedical Sciences, Louisiana State University, School of Veterinary Medicine, Baton Rouge, LA, United States; ^2^McGovern Institute for Brain Research, Department of Brain and Cognitive Sciences, Massachusetts Institute of Technology, Cambridge, MA, United States; ^3^Stanley Center for Psychiatric Research, Broad Institute of MIT and Harvard, Cambridge, MA, United States; ^4^Department of Genetic and Behavioral Neuroscience, Gunma University, Graduate School of Medicine, Maebashi, Japan

**Keywords:** VGAT, critical period, somatosensory, thalamus, laser-scanning photostimulation

## Abstract

The thalamic reticular nucleus (TRN) is the main source of inhibition to the somatosensory thalamus (ventrobasal nucleus, VB) in mice. However, the functional topography and development of these projections with respect to the VB barreloids has been largely unexplored. In this respect, to assist in the study of these projections, we have utilized a vesicular gamma-aminobutryic acid (GABA) transporter (VGAT)-Venus transgenic mouse line to develop a brain slice preparation that enables the rapid identification of inhibitory neurons and projections. We demonstrate the utility of our *in vitro* brain slice preparation for physiologically mapping inhibitory reticulothalamic (RT) topography, using laser-scanning photostimulation via glutamate uncaging. Furthermore, we utilized this slice preparation to compare the development of excitatory and inhibitory projections to VB. We found that excitatory projections to the barreloids, created by ascending projections from the brain stem, develop by postnatal day 2–3 (P2–P3). By contrast, inhibitory projections to the barreloids lag ~5 days behind excitatory projections to the barreloids, developing by P7–P8. We probed this lag in inhibitory projection development through early postnatal whisker lesions. We found that in whisker-lesioned animals, the development of inhibitory projections to the barreloids closed by P4, in register with that of the excitatory projections to the barreloids. Our findings demonstrate both developmental and topographic organizational features of the RT projection to the VB barreloids, whose mechanisms can now be further examined utilizing the VGAT-Venus mouse slice preparation that we have characterized.

## Introduction

**Figure 1:**
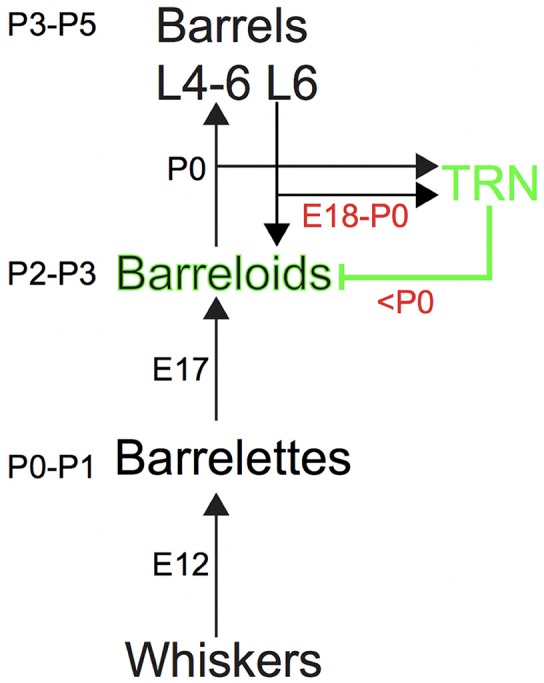
The somatosensory pathway from the whiskers to the barrel cortex in mice. Ascending and descending excitatory projections are illustrated by black arrows, whereas descending inhibitory projections from the thalamic reticular nucleus (TRN) are illustrated by the green line, respectively. Intrinsic inhibitory circuits are not illustrated. Each number (e.g., P3–P5 or E12) denotes the developmental age for establishing the brainstem barrelettes, thalamic barreloids and barrel cortex and the development of ascending and descending axons that reach the target station.

The thalamus represents an ideal structure to assess aspects of developmental plasticity of both excitatory and inhibitory projection systems. The thalamus is the obligate neural structure conveying sensory information to the cortex (Sherman and Guillery, 2002; Jones, 2007; Erzurumlu and Gaspar, 2012; Imaizumi and Lee, 2014) and receives feedforward excitatory projections from subthalamic structures and feedback excitatory corticothalamic (CT) projections from neurons in layer 6 of the neocortex (Sherman, 2016; Figure 1). In addition, the thalamus receives feedback inhibitory reticulothalamic (RT) projections from the thalamic reticular nucleus (TRN), which along with the zona incerta, are sources of inhibition to the ventrobasal nucleus (VB) in mice that lack local VB interneurons (Guillery and Harting, 2003; Pinault, 2004; Lam and Sherman, 2005; Sherman, 2016; Figure 1). Thus, the thalamus integrates excitatory and inhibitory projections in a thalamo-cortico-thalamic loop.

Despite its functional importance, structural plasticity in the thalamus has been much less appreciated than in the neocortex. Moreover, most ascending projections from the brainstem and thalamocortical (TC) projections are excitatory, such that studies of developmental plasticity in the thalamus have focused similarly on those excitatory projections. Interestingly, local inhibitory synapses can also be altered with excitatory synapses in developmental plasticity of the neocortex (Froemke, 2015). However, it remains unclear whether long-range inhibitory projections are similarly altered.

Even the seemingly rigid topographic maps of sensory space are amenable to structural plasticity in the developing nervous system (Sur and Leamey, 2001; Winer et al., 2004; Schreiner and Winer, 2007; Espinosa and Stryker, 2012). The well-defined whisker barrel maps in the primary somatosensory barrel cortex have demonstrated experience-dependent structural plasticity of developing TC projections (Inan and Crair, 2007; Lokmane and Garel, 2014). The mechanisms underlying such remodeling of excitatory projections involve the activity-dependent strengthening and pruning of excitatory synapses. By comparison, similar structural plasticity of inhibitory neural projections in the thalamus has not been intensively investigated, in part due to the lack of amenable preparations for investigating such changes.

Here, we take advantage of a vesicular gamma-aminobutryic acid (GABA) transporter (VGAT)-Venus transgenic mouse line to examine the developmental organization of inhibitory projections from the TRN to the barreloid field in the somatosensory thalamus (the VB nucleus). We demonstrate the utility of this preparation for *in vitro* slice physiological recordings to map the topographic organization of inhibitory projections to thalamic barreloids identified online. In addition, we demonstrate the development and structural plasticity of inhibitory projections to the barreloids using this preparation. Overall, we demonstrate a new preparation for studying the organization, development and structural plasticity of inhibitory projections to the barreloid region of the somatosensory thalamus.

## Materials and Methods

### Slice Preparation

All experimental procedures were approved by the Institutional Animal Care and Use Committee (IACUC) of the Louisiana State University School of Veterinary Medicine and the Committee on Animal Care of the Massachusetts Institute of Technology. Live slices were prepared from VGAT-Venus transgenic mice at P11–P13. These transgenic mice express the Venus fluorescent protein (pCS2-Venus developed in the laboratory of Dr. Atsushi Miyawaki at RIKEN, Wako, Japan) in VGAT—positive neurons (mouse line developed and shared by Dr. Yuchio Yanagawa at Gunma University) and obtained from Dr. Janice R. Naegele at Wesleyan University (Nagai et al., 2002; Wang et al., 2009).

**Figure 2:**
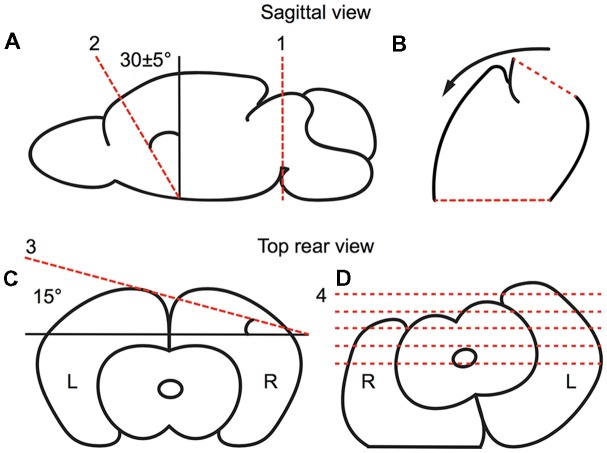
Preparation of the brain slice, as illustrated in a schematic drawing depicting the relevant blocking cuts. **(A,B)** Sagittal view of first blocking cut. **(A)** The brain was cut at the midbrain perpendicular to the midline (red broken line 1). Then, the brain was blocked at ~30° dorsoventrally (as illustrated by red broken line 2) from the rostral forebrain. **(B)** The blocked brain was then rested on the rostral blocked face (broken line 2 in **A**). **(C,D)** Rear view of second blocking cut. **(C)** From the rear, the blocked brain was blocked at 15° off the horizontal plane in the right cortex. **(D)** The blocked brain was then rested on the dorsal blocked surface and sectioned ventrodorsally.

Animals were deeply anesthetized under isoflurane. After decapitation, the brains were quickly removed and submerged in ice cold, oxygenated, artificial cerebral spinal fluid (ACSF; 125 mM NaCl, 3 mM KCl, 25 mM NaHCO_3_, 1.25 mM NaH_2_PO_4_, 1 mM MgCl_2_, 2 mM CaCl_2_, 25 mM d-glucose). Brains were blocked to preserve the thalamic barreloids (Figure 2). The blocking cuts were similar to the blocking angles for the auditory TC slice (Cruikshank et al., 2002; Lee and Sherman, 2008). A key distinction with our preparation to that previously described for the auditory TC slice is an initial 30° dorsoventral coronal blocking cut, followed by the 15° semi-horizontal blocking cut, which was found to well preserve the barreloid architecture (Figure 2). The blocked brains were glued on a stage with instant glue adhesive, ethyl cyanoacrylate (Elmers Krazy Glue, High Point, NC, USA), and then 500 μm thick sections were collected in ice-cold, oxygenated ACSF or sucrose-rich brain slice solution using a vibratome (World Precision Instruments, Sarasota, FL, USA; Lee et al., 2013). Collected slices were transferred to a holding chamber for 1 h at 32°C in oxygen-saturated ACSF and moved to a recording chamber perfused with oxygen-saturated aCSF at 32°C on a microscope stage (Siskiyou, Grants Pass, OR, USA).

### Slice Physiological Recordings and Laser-Scanning Photostimulation

Inhibitory projections from the TRN to the VB barreloids in the live slice preparation were imaged with a Retiga-EX camera (QImaging, Surrey, BC, Canada), using StreamPix5 (Norpix, Montreal, QC, Canada), mounted on an Olympus BX-51 upright microscope (Olympus, Tokyo, Japan) through a Chroma filter (41026; HQ495-30×, HQ 545-50 m, Q516 LP; Chroma, Rockingham, VT, USA; Lee and Imaizumi, 2013). Barreloids were identified and targeted on-line (Van Der Loos, 1976). Recordings were made from VB neurons at P11–13, when intrinsic membrane properties were relatively constant. To assist in isolating inhibitory currents, whole-cell recordings were made in voltage clamp mode using recording pipettes with tip resistances of 4–8 MΩ filled with a cesium intracellular solution (110 mM d-gluconic acid, 110 mM CsOH, 10 mM CsCl_2_, 1 mM CaCl_2_, 1 mM EGTA, 1 mM Mg-ATP, 10 mM HEPES, pH 7.3) to hold the cell at 0 mV.

Uncaging of glutamate by laser-scanning photostimulation was used to identify synaptic input locations in the TRN eliciting inhibitory postsynaptic currents (IPSCs) in the recorded barreloid cells. After patching, a recirculating ACSF bath containing 0.37 mM nitroindolinyl (NI)-caged glutamate (Sigma-Aldrich, St. Louis, MO, USA) was switched in place of the regular ACSF bath. Photolysis of the caged glutamate was made focally with a pulsed UV laser (DPSS Lasers Inc., Santa Clara, CA, USA). We used an 8 × 16 stimulation array with 80 μm spacing between stimulation spots. We repeated the mapping procedures 3–5 times for each neuron and averaged the resultant maps using the mapAnalysis and mapAverager programs in Ephus (Janelia Farms, Jupiter, FL, USA; Suter et al., 2010). Detailed procedures were described elsewhere (Lee and Imaizumi, 2013).

### Imaging VGAT-Venus in Barreloids in Fixed Tissue

After deep anesthesia under isoflurane, brains from juvenile VGAT-Venus mice (at P4–P14) were removed and blocked as described above (Figure 2; Cruikshank et al., 2002; Lee and Sherman, 2008), and fixed by submerging in 4% paraformaldehyde (PFA, Electron Microscopy Sciences, Hatfield, PA, USA) in 10 mM phosphate buffered saline (PBS, pH 7.3) for a few days (Lee et al., 2013). For cryoprotection, the brains were kept in 30% sucrose for 2–4 days. The blocked brains were mounted on OCT compound (Sakura Finetek, Tokyo, Japan) and sectioned at −20°C using a Leica cryostat (Leica Microsystems, Buffalo Grove, IL, USA). Sections (50 μm) were collected in 10 mM PBS (Lee et al., 2013) and coverslipped with Hardset Mounting Medium (Vector Labs). Images of VGAT-Venus expression in barreloids in the left hemisphere were captured using a Leica TCS SP2 confocal microscope (Leica Microsystems, Wetzlar, Germany) or a Retiga-EX camera mounted on an Olympus BX-51 upright microscope (Olympus, Shinjuku, Tokyo, Japan). Contrast of digitized images was enhanced in Fiji (Schindelin et al., 2012; biological image software based on ImageJ, National Institutes of Health, Bethesda, MD, USA) using same parameters.

### Vessicular Glutamate Transporter 2 (VGLUT2) Immunohistochemistry

For vessicular glutamate transporter 2 (VGLUT2) staining, sections were blocked with 10% normal goat serum (Vector Laboratories) and 0.5% Triton X-100 in PBS for 30 min, and incubated with 1:5,000 guinea pig anti-VGLUT2 antibody (Millipore, Bedford, MA, USA) overnight and 1:400 Alexa 568 conjugated goat anti-guinea pig IgG (Life Technologies, Carlsbad, CA, USA) for 2 h. Counterstaining was performed for nuclear staining using 1–2 μM To-Pro-3 Iodide (Life Technologies, Carlsbad, CA, USA) for 15 min. These sections were covered with Hardset Mounting Medium (Vector Laboratories, Burlingame, CA, USA) and imaged as described above.

### DiI Deposit

Brains were removed, blocked and fixed at P3, as described above. The blocked brains were mounted on a stage with instant glue adhesive and submerged in 10 mM PBS. Then, 200–300 μm sections were collected in 10 mM PBS using a vibratome (Ted Pella, Redding, CA, USA). Selected sections containing the TRN and the thalamic barreloid border (e.g., Figure 5A) were further fixed in 4% fresh PFA/PBS overnight. To estimate whether RT fibers were present at P3 in this slice preparation, small crystals of DiI (Life Technologies, Carlsbad, CA, USA) were manually deposited on the TRN in our slice preparations using an insect pin visually guided under a dissecting microscope (AmScope, Irvine, CA, USA). DiI enables fiber tracing in postmortem, fixed tissue (Chua et al., 1990; Ozaki and Wahlsten, 1992). These sections were incubated at 37°C in 4% PFA/PBS for a month and mounted on a slide using spacers (Electron Microscopy Sciences, Hatfield, PA, USA) and Fluoro-Gel mounting medium (Electron Microscopy Sciences) after washing in PBS. Images were captured by an Olympus Fluoview FV1000 confocal microscope (Olympus).

**Figure 3:**
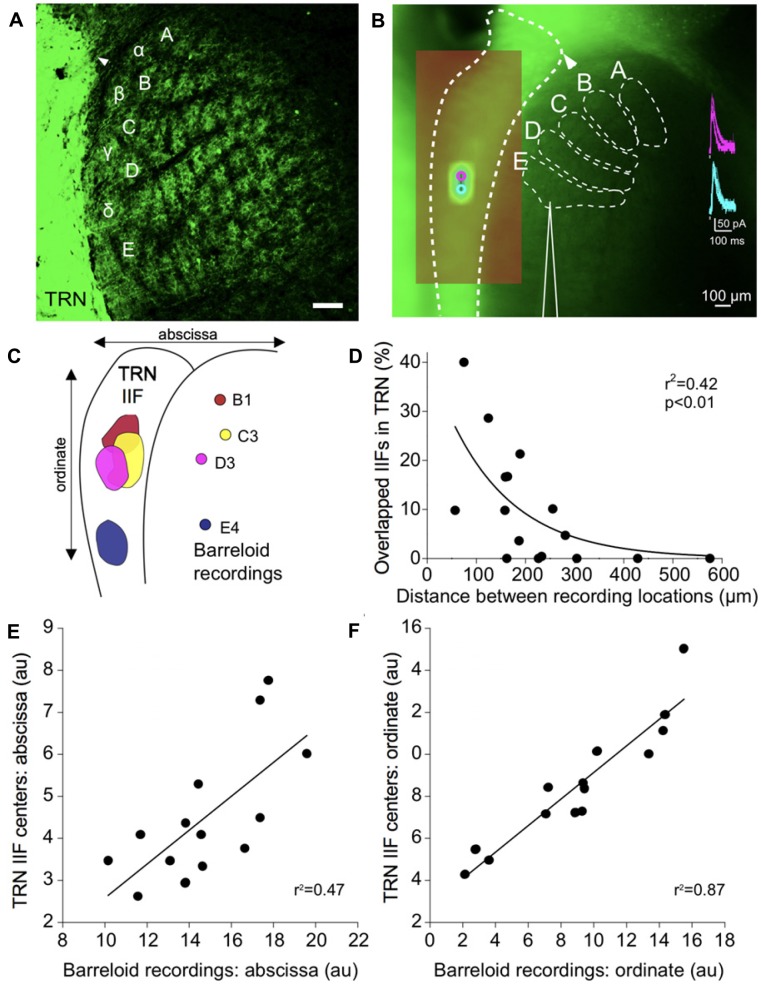
Functional topography of inhibitory projections to the barreloids. **(A)** Confocal image of inhibitory projections to the barreloids in a fixed brain slice (50 μm thickness) at P12. Arrowhead indicates alignment of the edge of the TRN with the *B* barreloid row. Scale: 100 μm. **(B)** Inhibitory projections to the barreloids as observed in the live *in vitro* brain slice (500 μm thickness). **(A–C)** Illustrates barreloid rows for reference. For identifying barreloids, the dorsal edge of the TRN (illustrated by the arrowhead) is a landmark, which is often aligned with the *B* barreloid row. Scale: 100 μm. (inset) Representative inhibitory postsynaptic currents elicited by photostimulation of the TRN. **(C)** Four representative inhibitory input fields (IIFs) in the TRN and their corresponding recording locations in thalamic barreloids from a same slice. Overlapped IIFs in TRN are 17% (B1-C3), 5% (B1-D3), 17% (C3-D3) and 0% with E4. **(D)** Significant relationship between overlapped IIFs in the TRN and distance between recording locations in thalamic barreloids. Sixteen recording pairs from the same slices (*n* = 4) were analyzed using non-linear regression. **(E)** Topographic organization between IIFs in TRN and recording locations in thalamic barreloids along the abscissa. Fourteen IIFs and the corresponding recording locations from all recorded neurons are aligned on a single map. IIF centers in TRN and recording locations in barreloids are significantly correlated in a linear regression. **(F)** Same as **(E)** analysis along the ordinate.

**Figure 4:**
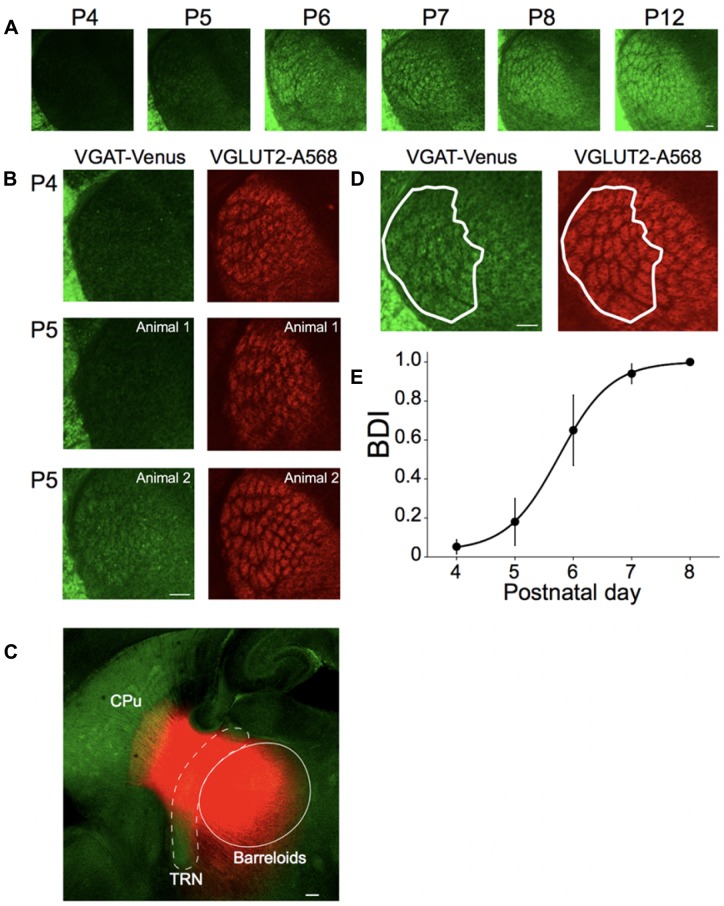
Postnatal development of inhibitory projections to the barreloids. **(A)** Development of inhibitory projections to the barreloids from P4 to P12. For illustration purposes, brightness and contrast of the images are enhanced. **(B)** Different development of inhibitory and excitatory projections to the barreloids. Left panels: inhibitory projections to the barreloids expressed by vesicular gamma-aminobutryic acid (GABA) transporter (VGAT)-Venus. Right panels: excitatory projections to the barreloids expressed by vessicular glutamate transporter 2 (VGLUT2) with Alexa 568 (A568). At P5–6, development of inhibitory projections to the barreloids exhibit large individual variability, which is also evident in the large SEM of the barreloid development index BDI scores in **(E)**. BDI scores: 0 (top), 0 (middle) and 0.63 (bottom). **(C)** Existence of reticulothalamic (RT) projections before P4. Small crystals of DiI were placed on the TRN in fixed sections (200–300 μm) at P3 and incubated in 4% paraformaldehyde (PFA) at 37°C for a month. DiI Labeling show an exponential decrease in intensity in the thalamus and striatum, likely representing the edge of transport for the dye, or the termination of these fibers in this slice preparation. In addition, since the borders of the barreloids observed using VGAT are not discernable at P3, we only indicate an approximate area for the barreloids. More precise delineation with VGLUT2 staining was not feasible in the thick slice prior to DiI labeling. The approximate TRN and barreloid regions are illustrated by white broken line and white line, respectively. RT projections are red. CPu, caudate putamen. **(D)** Identification of inhibitory projections to the barreloids (VGAT-Venus) shared with excitatory projections to the projections to the barreloids (VGLUT2-A568). A white line illustrates barreloid border analyzed for BDI. BDI score: 1. This section was obtained from the brain at P7. **(E)** Establishment of inhibitory projections to the barreloids. BDI scores are plotted as a function of postnatal day (mean ± SEM). Sigmoidal fitting reaches a plateau at P7–P8, indicating that inhibitory projections to the barreloids are established by P7–8. Number of animals: *n* = 3 (P4), *n* = 5 (P5), *n* = 3 (P6), *n* = 6 (P7) and *n* = 3 (P8). Scale bars in each panel: 100 μm **(A–D)**.

**Figure 5:**
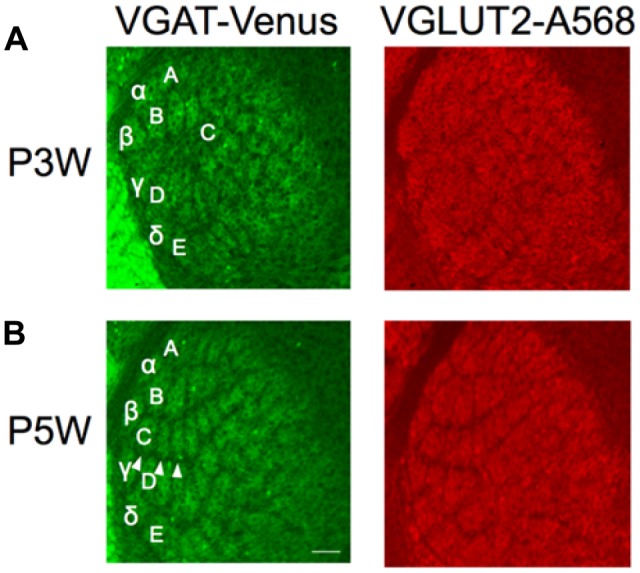
Structural plasticity of inhibitory projections to the barreloids. Whiskers and follicles in C1–C3 barreloids were removed at P3 (P3W) or P5 (P5W). Structural organization of the barreloids was assessed at P10. C1–C3 barreloids are missing following deafferentation at P3W **(A)**, but not at P5W (**B**; illustrated by white arrow heads). Left panels: barreloids labeled by VGAT-Venus. Right panels: barreloids labeled by VGLUT2 with Alexa 568 (A568). Letters and Greek symbols indicate barreloid rows.

### Whisker Lesion

Pups were anesthetized by inhalation of isoflurane (1%–5%). Before the surgery, meloxicam (1 mg/kg) was injected subcutaneously. Under a dissection microscope, whiskers in *C*1-*C*3 on the right side of the snout were carefully plucked out using a pair of sharp tweezers (Takeuchi et al., 2014), and these whisker follicles were surgically removed using a pair of sharp tweezers after making small incisions along the *C* barreloid row at P2–P3 or P5, respectively. Whiskers in these animals were observed to confirm no whisker growth before sacrificing animals. At P10, these animals were deeply anesthetized under isoflurane and fixed by 4% PFA/PBS. The brains were collected, postfixed, cryoprotected, sectioned, processed and imaged, as described above.

### Analysis

For *offline* analysis of electrophysiological recording locations, we identified each barreloid based on the position of the dorsal edge of the TRN (illustrated by an arrow head in Figures 3, 4), which is usually aligned with *B* barreloid row. However, four recording locations could not be unambiguously identified due to technical problems with our alignment. The inhibitory input field (IIF) was defined as the region of the TRN that elicited responses from the recorded VB neuron. This was estimated as pixel numbers using Fiji as follows:

$$\frac{overlapped\;IIF\;(pixels)}{sum\;of\;two\;IIFs\;(pixels)}\times 100\;(\%)$$

Distance between locations in the barreloids was estimated as pixel numbers in a linear measure using Fiji (Schindelin et al., 2012). To understand the relationship between overlapped IIFs in the TRN and the distance of recording locations in barreloids, we performed a non-linear regression analysis using an exponential decay model (Figure 3D). To assess topographic organization between IIFs in the TRN and recording locations in barreloids, IIF centers and recording locations were collapsed along an abscissa (Figure 3E) or an ordinate (Figure 3F), respectively, and a linear regression analysis was performed separately.

To quantify development of inhibitory projections to the barreloids, we introduce a barreloid development index (BDI). VGAT-Venus labeled barreloids and VGLUT2 labeled barreloids were scored by identifying shared barreloids. The BDI indicates when barreloids were labeled by both VGAT and VGLUT2:

$$\frac{the\;number\;of\;VGAT\;barreloids}{the\;number\;of\;VGLUT2\;barreloids}$$

BDI indices of zero indicate that no VGAT-labeled barreloids are found, while indicies near one indicate complete overlap of VGAT-labeled and VGLUT2-labeled barreloids. We quantified BDI in two to three sections from each brain, and the mean BDI score from each brain was plotted in Figure 4D. Complete barreloids appear only in a few sections at P8 or older animals. Raw data from all experiments are freely available for sharing upon request.

## Results

A major problem with studying barreloids in the somatosensory thalamus is the technical difficulty with accurately and unambiguously delineating the barreloid architecture. To solve this problem, we developed a slice preparation (Figure 2) that enables the straightforward identification of each barreloid. Our preparation takes advantage of transgenic-labeling of VGAT-positive inhibitory neurons, i.e., the VGAT-Venus mouse line (Wang et al., 2009). VGAT-Venus mice have been widely used to identify inhibitory neurons with Venus-labeled neuronal somata in various studies (Inada et al., 2011; Arami et al., 2013; Henderson et al., 2014; Bolton et al., 2015; Lee et al., 2015a,b). In addition, Venus is expressed in inhibitory axonal projections (Saito et al., 2015). We fully utilized these Venus-labeled projections from the TRN to understand synaptic and structural properties of the RT inhibitory projections. The structure of thalamic barreloids in a fixed slice is unambiguously delineated by Venus labeled RT projections from the TRN, since there are practically no intrinsic local inhibitory neurons in the rodent somatosensory thalamus (Cox et al., 1996; Guillery and Harting, 2003; Figure 3A). Using this preparation, each barreloid can also be easily identified in a live brain slice, for targeted physiological recordings from specific barreloids (Figure 3B).

### Structural Basis of Topographic Projections

To physiologically map the RT projections to specific barreloids, we made whole-cell recordings from barreloid neurons in an *in vitro* brain slice preparation. We confirmed the recording locations in each barreloid by offline examinations of the recording pipette placement. The inhibitory projections of the TRN were assessed by uncaging glutamate using laser-scanning photostimulation while making whole-cell recordings from a neuron in an identified barreloid (Lee et al., 2013). To isolate inhibitory currents, the resting membrane potentials of neurons were held at 0 mV using a cesium intracellular solution. Recorded barreloid neurons exhibited IPSCs responding to photostimulation of neurons within the TRN (Figure 3B inset). For all recorded barreloid neurons, the projection origin in the TRN was localized to 1–2 stimulation spots (80 μm separation between adjacent stimulation spots; Figure 3B inset). These stimulation loci were generally elongated along the long-axis of the TRN. Thus, these 1–2 activated stimulation spots in the TRN constitute the inhibitory TRN IIF of each barreloid neuron (see “Materials and Methods” section).

As an example, four recorded cells in B1, C3, D3 and E4 barreloids from the same live slice preparation received inhibitory RT input from topographically segregated but overlapped IIFs (5%–17%) in TRN (Figure 3C). However, no overlapped IIF was found when the recording locations were sufficiently separated, such as in three barreloids and the E4 barreloid (Figure 3C). Overlapped IIFs in the TRN vary from 0% to 40%, which are significantly and inversely correlated with distance between recording locations: the shorter the distance between recording locations in VGAT-labeled barreloids, the more the IIFs overlapped in the TRN (Figure 3D). We decomposed 14 TRN IIFs in a single map. Similar to that previously reported for projections from the TRN and somatosensory thalamus (Lam and Sherman, 2005, 2011; Lam et al., 2006), we found a topographic organization between IIF centers in the TRN and recording locations in thalamic barreloids (Figures 3E,F).

### Development of Inhibitory Projections to the Barreloids

Brainstem barrelettes, thalamic barreloids and cortical barrels are defined by functional clusters of ascending excitatory axons. We distinguished the VGAT-Venus labeled barreloids formed by inhibitory RT projections from those formed by feedforward excitatory projections using immunohistochemical labeling for VGLUT2, which is expressed on the ascending excitatory terminals.

The excitatory projections to the barrelette, barreloid and barrel structures are consecutively established by postnatal day 0–1 (P0–P1), P2–P3 and P3–P5, respectively, which follows the arrival of ascending excitatory axons from the lower to the higher station by embryonic day 12 (E12), E17, and P0, respectively (Ma, 1993; Erzurumlu and Gaspar, 2012; Mizuno et al., 2014; Takeuchi et al., 2014; Yamasaki et al., 2014; Figure 1). However, inhibitory projections to the barreloids are formed by feedback inhibitory RT axons from the TRN that receives collateral excitatory CT input from layer 6 of the barrel cortex (Figure 1). Ascending excitatory axons from the brainstem developmentally reach the thalamus by E17 (Erzurumlu and Gaspar, 2012), whereas descending CT as well as RT axons reach the thalamus at E18–E19 (Mitrofanis and Baker, 1993; Jacobs et al., 2007; Grant et al., 2012; Figure 1). It is also generally accepted that inhibitory circuits in the central sensory system developmentally lag behind excitatory circuits (Chang et al., 2005; Tao and Poo, 2005; Dorrn et al., 2010). Thus, we hypothesized that development of the inhibitory projections to the barreloids also lags behind that of the excitatory projections to the barreloids.

To test this hypothesis, first, we examined the development of inhibitory projections to the barreloids at different postnatal developmental periods (P4–P12) in fixed brain slices and quantified their development, as described below (Figure 4A). At P4, the outline of barreloids is formed, but VGAT-labeled barreloids are not yet visible. At P5–P6, individual VGAT-labeled barreloids are more visible. By P7–P8, VGAT-labeled barreloids clearly separated by septa are recognizable. At P12, the fully formed structure of the VGAT-labeled barreloids is clearly evident.

To confirm this developmental delay for the establishment of inhibitory projections to the barreloids compared to excitatory projections to the barreloids, we immunohistochemically stained for VGLUT2 in the developing brain at P4–P8. VGLUT2 is expressed in the ascending excitatory axonal projections from the brainstem (Kivrak and Erzurumlu, 2013). Whereas excitatory projections to the barreloids are established by P4 (Figures 1, 4B), inhibitory projections to the barreloids are not yet fully developed at P4 and exhibits individual variability at P5 during development (Figure 4B). Previous studies in the rat have reported that RT projections already exist before birth, but are immature and not fully functional until the second postnatal week (De Biasi et al., 1996, 1997; Figure 1). However, it is not clear whether RT projections also exist in the mouse even at P4 from our preparations. We investigated the existence of RT projections before P4 (before the appearance of VGAT-labeled barreloids). We placed a fluorescent lipophilic indocarbocyanine orange-red dye, DiI, in the TRN in fixed brain slices at P3 and found labeled fibers that emanated from the deposit site toward the region of VB and the striatum (Figure 4C). These fibers were consistent with the suggested early presence of RT projections from prior studies (De Biasi et al., 1996, 1997), although it is also likely that labeling of TC and/or CT fibers also contributed to the observed labeling.

To quantify the different developmental time course of excitatory and inhibitory projections to the barreloids, we identified the number of VGAT-labeled barreloids shared with VGLUT2-labeled barreloids at P4–P8 (Figure 4D). BDI (see “Materials and Methods” section) varies from zero (a ratio between identical inhibitory and excitatory projections to the barreloids) indicating no developed inhibitory projections to the barreloid to one, indicating a full establishment of inhibitory projections to the barreloids. A sigmoidal fitting of BDI scores reaches a plateau by P7–P8 (Figure 4E), suggesting that inhibitory projections to the barreloids are established by P7–P8 and lag 5 days behind excitatory projections to the barreloids. This is not an artifact caused by slow expression of VGAT-Venus because VGAT-Venus is already expressed in cell bodies as well as neurites at P0 (Inada et al., 2011).

### Critical Period of Structural Plasticity in Inhibitory Projections to the Barreloids

We next sought to understand structural plasticity of the inhibitory projections to the barreloids. It has been well documented that the critical period of structural plasticity of the thalamic excitatory projections to the barreloids and the barrel cortex closes by P4 (Yamakado, 1999; Erzurumlu and Gaspar, 2012). This period is after the establishment of excitatory projections to the barreloids (P2–P3) and during the establishment of the barrel cortex (P3–P5; Figure 1). Given the slow development of inhibitory projections to the barreloids (P7–P8; Figure 4), the critical period of structural plasticity in the inhibitory projections to the barreloids might extend beyond P4, similar to that found in the visual system, where the critical period of inhibitory neurons in ocular dominance plasticity of the mouse primary visual cortex lags behind that of excitatory neurons (Gandhi et al., 2008).

Here, we lesioned whisker follicles in the C row (C1, C2 and C3) on the right side of the snout at P2–3 or P5, respectively, in separate animals. We assessed the outcome of barreloid structure following whisker lesions by examining the barreloid architecture at P10. When the whiskers were lesioned at P3, the original thalamic area of the excitatory and inhibitory inputs to C1–C3 barreloids was replaced by D barreloids (Figure 5A). We then tested whether similar structural plasticity occurs by the same whisker lesions at P5. Based on our finding that the developmental period of inhibitory projections to the barreloids lagged behind that of the excitatory projections (Figure 4D), we posited that the critical period for the inhibitory projections might also lag behind that of the excitatory projections. However, we found that whisker lesions at P5 did not result in structural plasticity for either the excitatory or inhibitory projections to the barreloids (Figure 5B). Thus, we found a similar critical period prior to P5 for both the excitatory and inhibitory projections to the barreloids.

## Discussion

In this study, we developed a preparation that preserves inhibitory projections to the barreloids in a live slice for investigating functional topography. Unlike the barrel cortex, the barreloids are much less appreciated as a model system due to the difficulty of delineation. However, by using VGAT-Venus transgenic mice, we were able to capitalize on the rapid and unambiguous visualization of barreloid architecture in both live and postmortem slices.

### Barreloid Basis of Topographic Organization

Feedback RT projections are topographically organized (Lam and Sherman, 2005, 2011; Lam et al., 2006). Since our preparations allowed us to identify each barreloid online using epifluorescent microscopy, we extended previous studies of RT architecture that were unable to assign recording location to specific barreloids (Lam et al., 2006). We found that different barreloids have overlapped IIFs in TRN, potentially explained by the previous finding that individual TRN neurons have axon terminal bundles in more than one barreloid (Cox et al., 1996). Thus, these divergent projections from the TRN match the convergent projections to the barreloids in our results. The topography of RT barreloid projections was well preserved from recordings in single sections. However, when data were combined from separate experiments, this barreloid projection topography was less (yet highly significant statistically) preserved, perhaps due to different barreloid size and alignment artifacts across different animals. In the current study, we made recordings from neurons in up to four separate barreloids in a single slice preparation.

Future studies can utilize this preparation to understand the topographic organization within a single barreloid as well as along a single barreloid row or column. Moreover, the ability to identify and target specific barreloids for *in vitro* recordings can greatly simplify future studies of whisker sensation that combine slice physiology with *in vivo* experimentation.

### Delayed Development of Inhibitory Projection to the Barreloids

A surprising finding from our study is the delayed development of inhibitory projections to the barreloids compared to that of excitatory projections to the barreloids. The barrel system (e.g., the excitatory projections to the barreloids and the barrel cortex) is formed a few days after the arrival of ascending axons (Figure 1; Erzurumlu and Gaspar, 2012). Both excitatory axons from the brainstem and inhibitory axons from the TRN arrive at the thalamus before birth (E17 for excitatory axons and E18–E19 for inhibitory axons; Erzurumlu and Gaspar, 2012; Grant et al., 2012). Whereas the excitatory projections to the barreloids are formed at P2–P3, the inhibitory projections to the barreloids are formed at P7–P8, which lags 5 days behind that of the excitatory projections to the barreloids. This delay in the formation of the inhibitory projections to the barreloids cannot be simply accounted for by different axonal arrival time (Figure 1; Erzurumlu and Gaspar, 2012).

Development and maturation of inhibitory circuits in sensory systems lags behind that of the excitatory circuits (Chang et al., 2005; Tao and Poo, 2005; Dorrn et al., 2010). Indeed, prior ultrastructural studies of the developing RT projection to VB indicate that GABAergic synaptic terminals are present, but immature at birth and only reach full maturity by the second postnatal week in rats (De Biasi et al., 1996, 1997). Therefore, additional mechanisms likely account for delayed development of the inhibitory projections to the barreloids. One possibility is feedback excitatory input from cortical layer 6. Layer 6 CT projections reach the barreloids as early as E18 (Jacobs et al., 2007), but take longer to fully innervate the barreloids between P2 and P6 (Grant et al., 2012). The later time of this period (P5–P6) roughly matches the developmental period of the inhibitory projections to the barreloids. Perhaps, during this period, a balance between excitation and inhibitory inputs can be established for developing feedback projections (Froemke, 2015). Interestingly though, cortical layer 6 receives direct TC projections (Wimmer et al., 2010; Lee et al., 2012; Lee and Imaizumi, 2013), which form barrel-like fields in layer 6a, termed infrabarrels (Crandall et al., 2017). In the future, it will be necessary to examine how these infrabarrel layer 6 neurons affect development and formation of the inhibitory projections to the barreloids.

### Fixed Critical Period of Structural Plasticity in Inhibitory Projections to the Barreloids

Another surprising finding from this study is the fixed critical period of structural plasticity in the inhibitory projections to the barreloids. It has been well documented that the critical period of structural plasticity in the excitatory projections to the barreloids closes by P4 (Yamakado, 1999; Erzurumlu and Gaspar, 2012). In our study, whisker lesions before P4 resulted in structural plasticity both in excitatory and inhibitory projections to the barreloids (Figure 5). The same procedure was performed at P5 after the closure of the excitatory critical period but still during the development period of inhibitory projections to the barreloids. We found that the critical period of structural plasticity in the inhibitory projections to the barreloids had already closed despite the delayed development of the inhibitory projections to the barreloids. This suggests that the critical period of structural plasticity closes at the same time for both the excitatory and inhibitory projections to the barreloids and that delayed development of the inhibitory projections to the barreloids does not affect timing of critical period closure.

The molecular mechanisms underlying the critical period changes appear to be in part dependent on NMDA receptors, in particular NR2B, which affects barreloid development and the critical period closure of structural plasticity: early or delayed development corresponds to early or late closure of the critical period, respectively (Yamasaki et al., 2014). However, other NMDA receptors, NR2A and NR2D appear less important in this regard (Lu et al., 2001). It remains to be determined whether analogous effects are mediated through different GABA receptor subtypes, or other neurotransmitter receptors. These receptors are known to exhibit a developmental switch in the immature thalamus (Peden et al., 2008).

Physiologically, RT projections are able to elicit relatively weak responses at ages up through P5, whereas afterwards the strength of inhibitory responses increases dramatically (Evrad and Ropert, 2009). Thus, it is likely, as suggested from prior ultrastructural studies, that weak inhibitory RT to VB barreloid connections are present to be refined through signaling in the same critical window (De Biasi et al., 1996, 1997). Alternatively, as a group, thalamic reticular neurons are composed of chemically distinct subtypes, among which several express the calcium binding protein, calretinin (Lizier et al., 1997), which has also been shown to be poorly labeled in VGAT-Venus mice (Uematsu et al., 2008). Thus, a subset of RT projections may be present earlier, but not revealed in our VGAT-Venus preparation.

Potential insight into the formation of inhibitory projections to the projections to the barreloids may be drawn from related studies of the critical period of ocular dominance plasticity in the primary visual cortex, where a number of neural mechanisms underlying ocular dominance plasticity have been proposed (Hensch, 2005; Levelt and Hübener, 2012). Local inhibitory neurons may undergo structural and synaptic refinement, largely to regulate excitatory synaptic inputs. More recent studies have also proposed a contribution of microglia to synaptic pruning of excitatory projections (Schafer et al., 2012, 2013). Overall though, the focus is generally on the development of excitatory projections. And, it remains to understand whether similar synaptic pruning mechanisms underlie development, structural plasticity, and critical period of inhibitory projections.

### Perspectives

Our study raises several questions regarding the inhibitory projections to the barreloids that can be addressed using our slice preparation in the VGAT-Venus transgenic mouse line: canonical topographic organization, effects of cortical layer 6 on the development of inhibitory projection to the barreloids, and temporal pattern of synaptic pruning on inhibitory projections. In the future, it is also possible to observe excitatory and inhibitory synaptic projection-interactions with excitatory axons (e.g., from the brainstem or cortical layer 6) by crossing with relevant transgenic mouse lines. Such detailed studies should shed light not only on the development and structural plasticity of the barreloids but also their relevance to the neural mechanisms underlying related psychiatric disorders.

## Author Contributions

KI designed the experiments. KI and CL conducted the experiments and analyzed the data. YY developed and provided the VGAT-Venus mouse line. KI, GF and CL drafted and edited the manuscript.

## Conflict of Interest Statement

The authors declare that the research was conducted in the absence of any commercial or financial relationships that could be construed as a potential conflict of interest.
